# Married Life

**Published:** 1878-03

**Authors:** 


### MARRIED LIFE.

Married life has its trials and its sorrows. Tempers may prove incompatible, and call for forbearance. Fortune may be chary of its favors, and enforce self-denial. Children may be ungrateful, and sting the poor heart that has pillowed them. Sickness may come, and haunt a household for years. But ask the poor man, struggling along with his debts, and the weary woman, toiling early and late, accomplishing the ruin of her beauty and her buoyancy, if they would be placed apart, could competence be given them, and all their trials be brought to an end. The answer would be: "There is something sweeter in this companionship of suffering, than anything the world can offer from its storehouse of joys outside of it, and something which would make even severer trials than ours only iron bands to draw us more firmly together.

